# Downregulation of FXYD2 Is Associated with Poor Prognosis and Increased Regulatory T Cell Infiltration in Clear Cell Renal Cell Carcinoma

**DOI:** 10.1155/2022/4946197

**Published:** 2022-10-19

**Authors:** Zedan Zhang, Yanlin Tang, Lei Li, Wuping Yang, Yawei Xu, Jingcheng Zhou, Kaifang Ma, Kenan Zhang, Hongkai Zhuang, Yanqing Gong, Kan Gong

**Affiliations:** ^1^Department of Urology, Peking University First Hospital, Beijing, China; ^2^Hereditary Kidney Cancer Research Center, Peking University First Hospital, Beijing, China; ^3^Institute of Urology, Peking University, Beijing, China; ^4^National Urological Cancer Center, Beijing, China; ^5^Shantou University Medical College, Shantou, China; ^6^Department of Hepatobiliary Surgery, Sun Yat-sen Memorial Hospital, Sun Yat-sen University, Guangzhou, China

## Abstract

**Background:**

FXYD2, a gene coding for the *γ* subunit of Na^+^/K^+^-ATPase, was demonstrated to involve in carcinogenesis recently. However, the specific role of FXYD2 in clear cell renal cell carcinoma (ccRCC) remains unknown. The current study was conducted to investigate the expression, biological function, and potentially immune-related mechanisms of FXYD2 in ccRCC. *Materials and methods*. The data from TCGA-KIRC, ICGC, GEO, Oncomine, ArrayExpress, TIMER, HPA datasets, and our clinical samples were used to determine and validate the expression level, prognostic roles, and potentially immune-related mechanisms in ccRCC. Cell function assays were performed to investigate the biological role of FXYD2 in vitro.

**Results:**

FXYD2 was identified to be downregulated in ccRCC tissue compared to normal tissue, which was confirmed by our RT-PCR, WB, and IHC analyses. Kaplan-Meier survival analysis and Cox regression analysis suggested that downregulated FXYD2 could independently predict poor survival of ccRCC patients. Through the ESTIMATE algorithm, ssGSEA algorithm, CIBERSORT algorithm, TIMER database, and our laboratory experiment, FXYD2 was found to correlate with the immune landscape, especially regulatory T cells (Treg), in ccRCC. Gain-of-function experiment revealed that FXYD2 could restrain cell proliferation, migration, and invasion in vitro. Functional enrichment analysis illustrated that TGF-*β*-SMAD2/3, Notch, and PI3K-Akt-mTOR signaling pathways may be potential signaling pathways of FXYD2 in ccRCC.

**Conclusions:**

Downregulation of FXYD2 is associated with ccRCC tumorigenesis, poor prognosis, and increased Treg infiltration in ccRCC, which may be related to TGF-*β*-SMAD2/3, Notch, and PI3K-Akt-mTOR signaling pathways. This will probably provide a novel prognostic marker and potential therapeutic target for ccRCC.

## 1. Introduction

Renal cell carcinoma accounts for 3.7% of cancer cases all around the world, and it is one of the 10 most common cancers [1]. The major component of this cancer is clear cell renal cell carcinoma (ccRCC), whose rate diagnosed as being metastatic stage at the first visit was unignorable, leading to the requirement of more effective diagnostic and prognostic markers [2]. With the development of high-throughput sequencing technology, gene expression is widely investigated and demonstrated that it can be utilized in dealing with cancers [3]. Multiple genes have been identified to be diagnostic and prognostic biomarkers in ccRCC, and some of them can be potentially regarded as therapeutic targets. However, few gene markers can be transferred into clinical usage, which indicates the necessity of further investigation [4–6].

Na^+^/K^+^-ATPase, also called sodium pump, is an oligomeric protein located in the plasma membrane of epithelial cells and transports three Na^+^ out of cells in exchange for two K^+^ into cells [7]. Abnormal expression of Na^+^/K^+^-ATPase correlates with the development and progression of different cancers, which is regarded as an important target for the development of anticancer drugs as it has a multifunctional signal transduction and plays a vital role in cell adhesion [8]. The outcome of Na^+^/K^+^-ATPase inhibitors can affect some downstream signaling pathways when binding to Na^+^/K^+^-ATPase, which can inhibit cell proliferation and induce apoptosis in cell and autophagy-mediated cell death [9, 10]. FXYD protein family contains a group of small transmembrane segment proteins modulating the properties of Na^+^/K^+^-ATPase to adjust the homeostasis of ion balance in a tissue-specific manner [11]. Recently, FXYD proteins have been discovered to participate in carcinogenesis [12]. Among this family, FXYD2, a gene coding for the *γ* subunit of Na^+^/K^+^-ATPase, was found to be abundantly expressed in kidney and pancreas. In the kidney, the *γ* subunit is expressed as two splice variants, termed the *γ*a and *γ*b subunit, which are primarily expressed in the thick ascending limb and induce reduction of the Na^+^/K^+^-ATPase affinity for sodium [13, 14]. It was also reported to be able to increase potassium antagonism of intracellular sodium binding, revealing an additional effect of *γ* subunit on intrinsic binding of potassium at cytoplasmic sites [15]. The Na^+^ activation of Na-K pump currents is inhibited by FXYD2 [16], which parallelly decreases Na^+^/K^+^-ATPase activity for Na^+^ and K^+^ [17]. However, the more specific physiological functions of Na^+^/K^+^-ATPase modulated by FXYD2 in the kidney remain more speculation. Induction of FXYD2 in cells of renal origin showed the reduced activity of Na^+^/K^+^-ATPase and the rate of cell division, suggesting FXYD2 could probably response to genotoxic stress [18]. Abnormal expression of FXYD2 was identified in chromophobe RCC and was regarded as a possible marker for differential diagnosis [19]. It was also revealed to be associated with growth of ovarian clear cell carcinoma and intraocular tumors, which represents as a potential therapeutic target [19, 20] . Nonetheless, existing evidence about the role of FXYD2 in tumors including ccRCC is quite limited. Therefore, the study of the expression and potential function of FXYD2 in ccRCC is of great significance for the discovery of new biomarkers for diagnosis and prognosis of ccRCC. Given that the inhibitors for Na^+^/K^+^-ATPase show antitumor effects and the intimate connection between FXYD2 and Na^+^/K^+^-ATPase, there is great potential to explore targeted drugs again FXYD2 to assist in the treatment of ccRCC.

CcRCC is generally managed with surgery but lacks potent therapeutic drugs due to its poor responsiveness to chemotherapies [21]. With a better understanding of immune biologics, novel immunotherapies are introduced and reported to induce notable benefits in the prognosis of ccRCC [22]. However, due to the heterogeneity of tumor and different drug response, the clinical uncertainty of immunotherapies restricts the expansion of current therapies and leads to an interest in discovering novel immune targets [23]. In the immune microenvironment of ccRCC, infiltration of regulatory T cells (Treg) was identified to be increased and they were associated with poor survival of ccRCC patients [24]. It was demonstrated that Treg could hamper the protective function of CD8^+^ T cells in ccRCC and prevent tumor cells from immune attack [25]. Besides, the uncontrolled Treg infiltration in ccRCC can contribute to the failure of therapy in the clinic [26, 27]. Instead, drugs modulating the development and differentiation of Treg were shown to have a great effect on ccRCC management with proven safety and efficacy [28, 29]. Therefore, a comprehensive cognition of the mechanism underlying infiltrating immune cells in the ccRCC microenvironment can assist in clinical management with immunotherapy and help discover novel therapeutic targets.

In the present study, we discussed the expression level and prognostic role of FXYD2 in ccRCC. Then, the relationship between FXYD2 expression and immune cell infiltration in ccRCC was explored through ESTIMATE and ssGSEA algorithm and TIMER database. Furthermore, GO and KEGG enrichment analyses were utilized to investigate the biological pathways through which FXYD2 affects ccRCC. Among these ways, novel biomarkers for ccRCC can be identified and potential biological pathways may be elucidated for the development of immunotherapy.

## 2. Materials and Methods

### 2.1. Public Dataset Acquisition

A few major public databases, such as TCGA (The Cancer Genome Atlas, http://cancergenome.nih.gov), ArrayExpress.(https://www.ebi.ac.uk/arrayexpress), GEO (Genome Expression Omnibus, https://www.ncbi.nlm.nih.gov/geo), Oncomine online database (http://www.oncomine.org), and ICGC (International Cancer Genome Consortium, https://dcc.icgc.org/), were adopted in our study. RNA-sequencing data of ccRCC were downloaded from TCGA-KIRC with the corresponding clinicopathological information, including 539 tumor and 72 normal subjects. Another microarray dataset containing clinical information (E-MTAB-3267, *n* = 59) was retrieved from ArrayExpress. Transcriptomic data in GSE40435 (*n* = 202) and GSE53757 (*n* = 144) were extracted from GEO database. Lenburg (*n* = 18), Gumz (*n* = 20), and Beroukhim (*n* = 81) were three microarray datasets of ccRCC obtained from Oncomine database. Gene expression profiles and clinical information of RCC patients (RECA-EU) were extracted from ICGC (*n* = 136). Besides, immunohistochemical (IHC) staining of 6 patients with or without renal cell adenocarcinoma was downloaded from The Human Protein Atlas Project (https://www.proteinatlas.org). The above data were all from public databases with ethics approval provided.

### 2.2. Clinical Sample Acquisition

This study was approved by the Biomedical Research Ethics Committee of Peking University First Hospital. 32 pairs of fresh matched ccRCC and paracancerous normal tissue samples were acquired from Peking University First Hospital, Beijing, China, which were snap-frozen in liquid nitrogen immediately for subsequent quantitative real-time polymerase chain reaction, western blot, and immunohistochemistry analysis. The written informed contents were confirmed from each patient involved in this study.

### 2.3. FXYD2 Expression Analysis in Public Datasets

To elucidate the expression of FXYD2 in ccRCC, R package limma [30] was utilized to perform differential expression analysis between tumor and normal tissues in above public cohorts. At the same time, transcriptome expression analysis for matched 72 pairs of tumor and corresponding adjacent normal tissues from TCGA provided further validation. The UALCAN online tool [31] based on TCGA database was used to show the expression of FXYD2 in different individual stage, tumor grade, nodal metastasis status, race, and gender. Moreover, immunohistochemistry images from The Human Protein Atlas (https://www.proteinatlas.org/) depicted the protein level of FXYD2 expression in cancer and normal tissue. The expression of FXYD2 in pan-cancer and corresponding normal tissues were also acquired from TIMER2.0 online database [32].

### 2.4. RNA Extraction and Quantitative Real-Time Polymerase Chain Reaction (RT-qPCR)

Total RNA isolation from clinical samples was performed using TRIzol reagent (Invitrogen, USA) according to the manufacturer's protocol. Centrifuge tubes and pipette tips were obtained from NEST Biotechnology Co. Ltd. (Wuxi, China). The purity and concentration of the RNA samples were determined by the A260-A280 nm ratio. Then, first-strand cDNA was synthesized through the reverse transcription method (Invitrogen, USA). RT-qPCR was carried out using SYBR Green Master Mix (Invitrogen, USA). Glyceraldehyde 3-phosphate dehydrogenase (GAPDH) was used as an internal control. The following primers were used: FXYD2, 5′-ATCCTCCTCAGTAAGTGGGGT-3′ (Forward) and 5′-CTTGGCAACTCCCGAAAGC-3′ (Reverse); FOXP3, 5′- GTGGCCCGGATGTGAGAAG-3′ (Forward) and 5′- GGAGCCCTTGTCGGATGATG-3′ (Reverse); IL2RA, 5′- GTGGGGACTGCTCACGTTC-3′ (Forward) and 5′- CCCGCTTTTTATTCTGCGGAA-3′ (Reverse); and GAPDH, 5′- GGAGCGAGATCCCTCCAAAAT-3' (Forward) and 5′- GGCTGTTGTCATACTTCTCATGG-3′ (Reverse).

### 2.5. Cell Lines and Cell Culture

786-O and OSRC2 ccRCC cell lines, obtained from American Type Culture Collection (Rockville, MD, USA), were used in our study and grown in DMEM (GIBCO, Carlsbad, USA) supplemented with 10% fetal bovine serum (FBS; Procell, Wuhan, China). FXYD2 overexpression plasmid was constructed by SyngenTech Company (SyngenTech Co. Ltd., Beijing, China). Lentivirus were constructed using corresponding vectors by Lipofectamine 3000 (Invitrogen, USA) according to the manufacturer's protocol. Two stable cell lines were established by lentivirus infection.

For gene knockdowns, two treated stable cell lines, 786-O and OSRC2 FXYD2 overexpression cell lines, were transfected with small interfering RNA (siRNA) infection including negative control using Lipofectamine 3000 (Invitrogen, USA) at 50 nM concentration according to the manufacturer's protocol for 1 day. Then, the cells were harvested for analyses 48 hrs after initial siRNA infection. The targeted sequences (SyngenTech Co. Ltd., Beijing, China) used were as follows: siFXYD2 #1, ACUAUGAGACCGUUCGCAATT; siFXYD2 3′UTR #1, UUGCGAACGGUCUCAUAGUTT; siFXYD2 #2, CAAUAAGAAGCGCAGGCAATT; siFXYD2 3′UTR #2, UUGCCUGCGCUUCUUAUUGTT; siFXYD2 #3, CAAUGAAGAUGAGCCGUAATT; and siFXYD2 3′UTR #3, UUACGGCUCAUCUUCAUUGTT.

### 2.6. Cell Proliferation Assay

The proliferation rate was determined by Cell Counting Kit-8 assay (CCK-8; KeyGen BioTECH, Jiangsu, China; LABLEAD, Beijing, China). The established cell lines were seeded in a 96-well plate with 1 × 10^3^ cells/well. Then, on days 0, 1, 2, 3, 4 and 5, the cells were incubated with CCK-8 for 2 hrs at 37 °C. The absorbance was measured at 450 nm using a microplate reader. The experiments were repeated at least three times.

### 2.7. Colony Formation Assay

The colony-formation ability of tumor cells was determined by colony formation assay. The established stable cell lines (786-O and OSRC2 FXYD2 overexpression cell lines) and corresponding siRNA-transfected cell lines were seeded into 6-well plates with a low density and incubated at 37 °C with 5% CO_2_ for 10-14 days. Then, the cells were washed with PBS, fixed with 4% paraformaldehyde for 15 min at room temperature and stained with 0.1% Crystal Violet Staining Solution.

### 2.8. Migration and Invasion Assay

For the migration assay, 3 × 10^4^ transfected 786-O cell lines and 4 × 10^4^ transfected OSRC2 cell lines were seeded into a upper chamber of a Transwell 24-well migration chamber (Costar, #3422, Corning) with 150 *μ*L serum-free DMEM. The lower chamber was filled with 600 *μ*L of 10% FBS-DEME as a chemoattractant. For the invasion assay, 100 *μ*L of Matrigel Basement Membrane Matrix as 1 : 8 dilution in serum-free DMEM (#354234, Corning) was added in the upper chamber and incubated at 37 °C for 2 hrs before use. After 24 hrs of incubation, cells migrated or invaded into the lower chamber were washed with PBS, fixed with 4% paraformaldehyde for 15 min and stained with 0.1% Crystal Violet Staining Solution for 15 min. Then, the cells on the lower surface were photographed by an inverted light microscope.

### 2.9. Western Blot (WB) Analysis

Protein of clinical tissue samples was extracted using the RIPA lysis buffer (Beyotime) with protease inhibitor (Beyotime). The BCA protein assay Kit (Invitrogen, USA) was used to quantitate total protein level. Then, the obtained protein samples (100ug) were separated by 12% SDS-PAGE and electrotransferred onto PVDF membranes (Millipore, USA), which were blocked with 5% skim milk and incubated with anti-FXYD2 (1 : 1000, Proteintech, 11198-1-AP) and anti-*β*actin (1 : 2500, Proteintech, 20536-1-AP) at 4 °C overnight. After incubated with peroxidase-conjugated goat anti-rabbit IgG (1 : 10000, ZSGB-BIO, ZB-2301), the membranes were visualized using Chemiluminescence (Merck Millipore, USA).

### 2.10. Immunohistochemistry (IHC) Analysis

The formalin-fixed paraffin-embedded tumor and normal sections were prepared for IHC analysis. According to manufacturer's instructions of Rabbit Two-Step Kit (Rabbit Polymer Assay System, ZSGB-BIO, PV-6001), after deparaffinization, rehydration, antigen retrieval, and endogenous peroxidase inhibition, the sections were incubated with rabbit antibody anti-FXYD2 (1 : 300, Affinity, DF9941) at 4 °C overnight. Then, horseradish peroxidase-conjugated goat anti-rabbit IgG polyclonal as secondary antibodies were used to incubate the sections. DAB Horseradish Peroxidase Color Development Kit (Beyotime, China) was used to detect FXYD2 expression under a light microscope, which was independently evaluated by two pathologists.

### 2.11. Prognostic Analysis of FXYD2 in ccRCC

Four prognostic indexes, such as overall survival (OS), progression-free survival (PFS), disease-specific survival (DSS), and disease-free survival (DFS), were cooperatively analyzed to illustrate the relationship between FXYD2 expression and patients' survival time using the corresponding information in the TCGA cohort. X-tile 3.6.1 software [33] was selected to provide an optimal cutoff point for dividing the expression of FXYD2 into high and low groups. Thereafter, survival analysis was conducted through R package “survminer” [34] with the results presented in form of Kaplan-Meier survival curves. Additional survival analysis of patients in the E-MTAB-3267 and ICGC cohorts were conducted for validation, while the five datasets of GEO and Oncomine databases cannot be used in validation due to their lack of survival time information. Then, the prognostic value of FXYD2 expression was challenged by univariate cox regression analysis together with the several clinicopathological features (age, gender, race, smoking, radiation, pharmaceutical intervention, tumor grade, pathologic stage, stage T, stage N, and stage M). Multivariate cox regression analysis was performed to uncover which value could independently predict ccRCC outcome. *P* value < 0.05 was regarded as significant.

### 2.12. Correlation Analysis between FXYD2 and Clinicopathological Features

The TCGA cohort was stratified into different groups according to the clinicopathological features and survival analysis was conducted, respectively, in each group to demonstrate the prognosis based on FXYD2 expression. The potential relation between FXYD2 expression and the prognosis of ccRCC patients was further investigated through analyzing FXYD2 expression and clinicopathological features using Chi-square test. *P* value < 0.05 was regarded as significant.

### 2.13. Analysis of FXYD2-Related Immune Cell Infiltration in ccRCC

ESTIMATE (Estimation of Stromal and Immune cells in Malignant Tumor tissue using Expression data) is an algorithm calculating immune score, stromal score, and tumor purity for conjecture of normal cells infiltration and tumor cellularity [35]. ESTIMATE algorithm was applied in TCGA, GSE40435, GSE57357, and E-MATB-3267 cohorts to depict the immune landscape related to FXYD2 expression in ccRCC. SsGSEA (single-sample Gene Set Enrichment Analysis) is a measure for investigation of the tumor-infiltrating immune cells together with their associated functional pathway information [36]. CIBERSORT (cell type identification by estimating relative subsets of RNA transcripts) is a computational approach for characterizing cell composition from complex tissue [37]. FXYD2-associated immune landscape in the TCGA cohort was analyzed through ssGSEA using R package “GSVA” [38] and CIBERSORT using R package “e1071,” “parallel,” and “preprocessCore.” Then, the same procedures were repeated in GSE40435, GSE57357, E-MATB-3267, and ICGC cohorts for validation. Regulatory T cells appeared to have great significance in most of cohorts. Therefore, the following analyses focused on the relation between FXYD2 expression and Treg infiltration level. Considering the relationship of immune microenvironment and ccRCC prognosis, patients in the TCGA cohort were divided into two groups, respectively, according to the level of immune score, stromal score, tumor purity, and Treg infiltration level utilizing X-tile 3.6.1. Survival analysis was conducted for each two groups and demonstrated by Kaplan-Meier survival curves. For a better understanding of the relation between FXYD2 and Treg, correlation analysis was conducted between FXYD2 expression and Treg infiltration level whose values were calculated using ssGSEA and CIBERSORT algorithm. Furthermore, the association between the gene markers of Treg and FXYD2 expression was explored through TIMER with or without purity adjustment. Since the correlation analysis indicated a negative relationship between FXYD2 expression and Treg infiltration level, the gene markers of T cell exhaustion were also examined.

### 2.14. Functional Enrichment Analysis

To conduct functional enrichment analysis, Pearson correlation analysis of the gene expression in TCGA cohort was firstly conducted to select the co-expression genes of FXYD2 according to the resulted coefficients (Pearson correlation coefficient > 0.4 or < −0.4, *P* value < 0.05). Then, functional enrichment analysis of these genes was performed in ConsensusPathDB (https://cpdb.molgen.mpg.de/), including GO (Gene ontology) and KEGG (Kyoto Encyclopedia of Genes and Genomes) analyses. GO is a community-based resource supplying data for functional annotation of genomic products in three aspects, biological process (BP), molecular function (MF), and cellular component (CC) [39]. KEGG serves as a database to connect gene expression with the molecular interaction network in cells for functional information in higher order [40]. Thereafter, the relationship between FXYD2 expression and the potential biological functions was elucidated and the enrichment scores of these biological functions were obtained using the R package “GSVA” [38].

### 2.15. Statistical Analysis

All statistical analyses were performed through R studio version 3.6.1. Continuous variables are expressed as mean ± SD. The Pearson *χ*^2^ test or Fisher's exact test was used to analyze the relationship between FXYD2 protein expression level and clinicopathological features.

The boxplot between tumor and normal groups was analyzed by Wilcoxon test. Survival curves were plotted using the Kaplan-Meier method, and Log-Rank test was applied to compare the difference between groups. Time-dependent receiver operating characteristic curve (tROC) analysis was performed, and the Uno's C-index was calculated using the “survivalROC” and “survcomp” package of R software, respectively. Graph constructions were achieved utilizing both R studio version 3.6.1 and GraphPad Prism 8.0.1. *P* value < 0.05 was regarded as significant for all statistical analyses.

## 3. Results

### 3.1. FXYD2 Expression Is Downregulated in ccRCC

**Figure 1:**
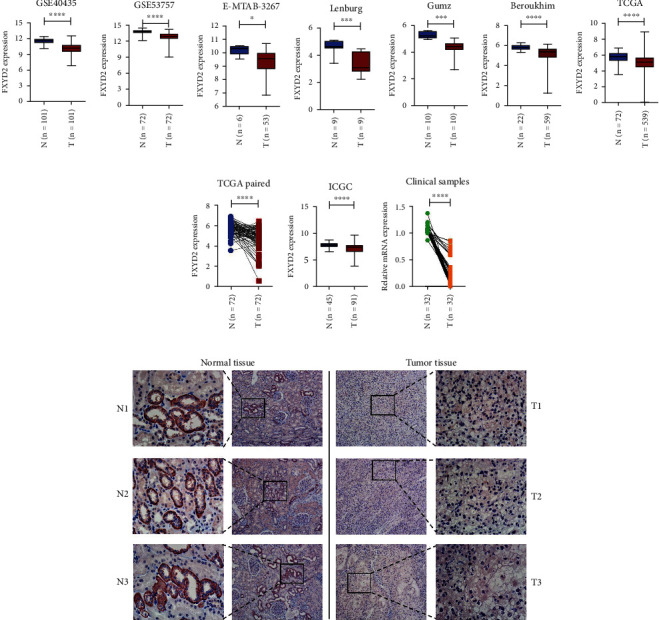
Differential expression analysis of FXYD2 in ccRCC and normal tissue. (a–g) (i) FXYD2 expression was downregulated in ccRCC tissue compared with normal tissue in GSE40435, GSE53757, E-MTAB-3267, Lenburg, Gumz, Beroukhim, TCGA, and ICGC cohort, respectively. (h) The paired expression pattern of the FXYD2 expression showed downregulated pattern in ccRCC tissue compared with adjacent normal tissue. (j) RT-qPCR analysis of ccRCC and normal tissue validated the difference of expression. (k) IHC analysis of ccRCC and normal tissue validated the difference of expression. CcRCC: clear cell renal cell carcinoma; TCGA: The Cancer Genome Atlas; ICGC: International Cancer Genome Consortium; RT-qPCR: quantitative real-time polymerase chain reaction; IHC: immunohistochemistry; ^∗^*P* < 0.05, ^∗∗^*P* < 0.01, ^∗∗∗^*P* < 0.001, and ^∗∗∗∗^*P* < 0.0001.

GEO datasets (GSE40435, GSE53757), E-MTAB-3267, and three Oncomine datasets (Lenburg, Gumz, and Beroukhim) all showed that FXYD2 mRNA expression was downregulated in ccRCC samples compared to paracancerous normal samples (*P* < 0.05) (Figures 1(a)–1(f)). In the TCGA cohort, FXYD2 mRNA expression was also downregulated in 539 ccRCC compared to 72 normal tissue (*P* < 0.0001) (Figures 1(g)–1(h)).

**Figure 2:**
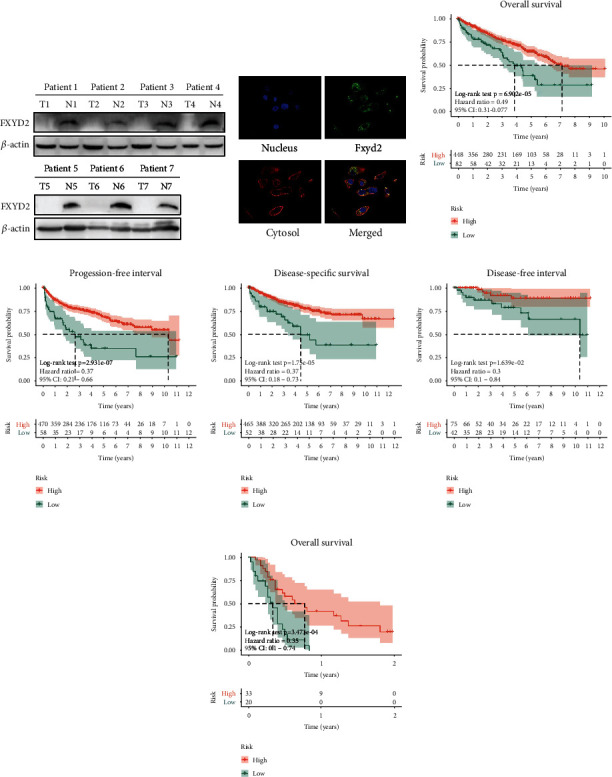
Western blot, subcellular location, and Kaplan-Meier survival analysis of FXYD2 in ccRCC. (a) Western blot analysis of FXYD2 from clinical samples showed downregulated expression in ccRCC tissue. (b) Subcellular location from The Human Protein Atlas showed FXYD2 proteins locate in cytoplasm. (c–f) CcRCC patients with low FXYD2 expression correlated with short-term overall survival, progression-free survival, disease-specific survival, and disease-free survival, respectively, in the TCGA cohort. (g) CcRCC patients with low FXYD2 expression correlated with short-term overall survival in the E-MTAB-3267 cohort.

72 matched pairs of ccRCC and adjacent normal tissues provided the same trend (*P* < 0.0001). In the ICGC cohort, FXYD2 mRNA expression was also downregulated in RCC samples compared with adjacent normal tissue (*P* < 0.0001) (Figure 1(i)). UALCAN online tools exhibited that the expression of FXYD2 mRNA was related to patient's tumor grade, cancer stages, nodal metastasis status, race, and gender (Figures S1(a)-S1(e)). IHC images from The Human Protein Atlas also verified the low FXYD2 expression level in ccRCC compared to the normal ones (Figures S1(f)–S1(g)). RT-PCR (Figure 1(j)), IHC (Figure 1(k)) and WB (Figure 2(a)) analysis using our clinical tissue samples also verified the transcription and translation level of FXYD2 expression, which all showed downregulated expression of FXYD2 in ccRCC compared to the normal tissue. Immunofluorescence images about the subcellular location from The Human Protein Atlas showed that FXYD2 favors cytoplasmic localization (Figure 2(b)).

### 3.2. Downregulated FXYD2 Is Associated with Progression and Poor Survival of ccRCC Patients

**Figure 3:**
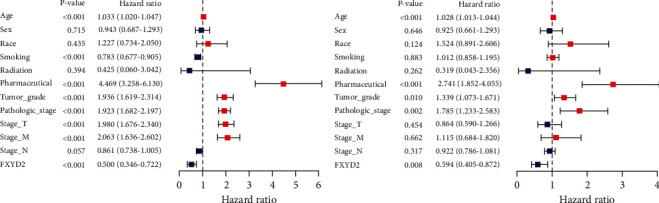
Independent prognostic analysis of FXYD2 in TCGA cohort. (a) Univariate cox regression analysis of FXYD2 in TCGA cohort. (b) Multivariate cox regression analysis of FXYD2 in TCGA cohort. Red square indicates HR larger than 1, while blue square indicates HR smaller than 1. HR: hazard ratio.

Totally, there are 539 tumor and 72 normal clinical samples from TCGA. Then, the survival time of the samples less than 10 days and duplicate samples were excluded. Therefore, there were 530, 517, 528, and 117 tumor samples with OS time, PFS time, DSS time, and DFS time, respectively. Kaplan-Meier survival analysis indicated that low-risk group had lower OS, PFS, DSS, and DFS (*P* < 0.0001, *P* < 0.0001, *P* < 0.0001, and *P* < 0.01, respectively) in the TCGA cohort (Figures 2(c)–2(f)). Validation of OS in the E-MTAB-4367 dataset delivered a similar result (Figure 2(g)). Time-dependent ROC analysis showed that AUC for 1-year, 2-year, 3-year, 4-year, and 5-year OS of the FXYD2 signature were 0.656, 0.611, 0.564, 0.600, and 0.563, respectively, (Figure S2(a)) (C − index = 0.588, 95% CI 0.538-0.637, *P* < 0.001). Besides, the survival analysis in the ICGC cohort revealed no significant relation between FXYD2 expression and patient prognosis (Figure S2(b)). This was most likely due to the confounding RCC patients in the ICGC cohort which includes not only ccRCC but also papillary RCC, chromophobe RCC, and others. There was no explicit illustration for patient classification on the homepage. Furthermore, the univariate and multivariate cox regression analyses were performed for the predictive role of FXYD2 in ccRCC. As the result demonstrated, FXYD2 expression, age, smoking, pharmaceutical intervention, tumor grade, pathologic stage, stage T, and stage M could predict the overall survival of ccRCC patients (Figure 3(a)). However, only FXYD2 expression, age, pharmaceutical intervention, tumor grade, and pathologic stage could independently predict the prognosis of ccRCC (Figure 3(b)). In this way, FXYD2 expression was proven to be an independent prognostic predictor that inversely correlated with the survival of ccRCC patients.

**Figure 4:**
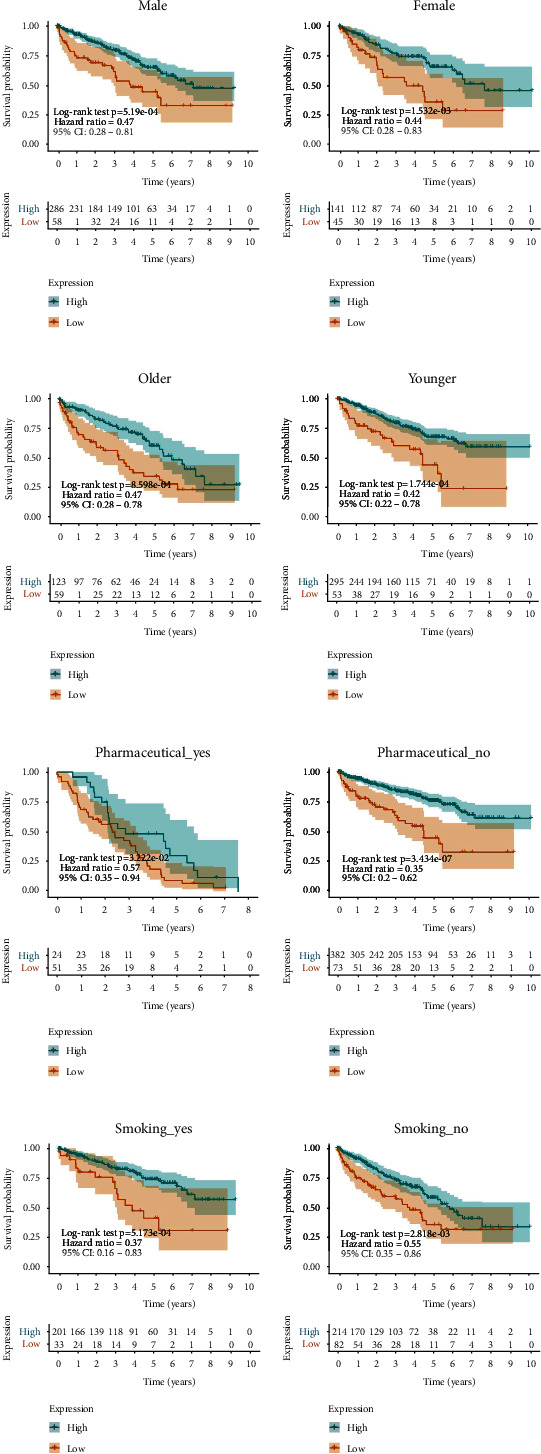
Kaplan-Meier survival analysis of FXYD2 concerning different clinicopathological factors. (a) Male and female ccRCC patients with low FXYD2 expression both had short-term overall survival. (b) Either patient more or less than 60 years old had short-term overall survival when their FXYD2 expression was low. (c) Either patient with or without pharmaceutical intervention had short-term overall survival when their FXYD2 expression was low. (d) Either smoking or nonsmoking patient had short-term overall survival when their FXYD2 expression was low.

Then, Chi-square test was performed between FXYD2 expression and different clinicopathological factors to elucidate the relationship of FXYD2 expression and clinicopathological features. As shown in Table 1, low FXYD2 expression was associated with high pathologic grade (*P* = 0.016) and high T stage (*P* = 0.001). Stratified survival analyses under different clinicopathological circumstances, including gender, age, with or without pharmaceutical intervention, with or without smoking, stage, grade, and with or without metastasis, indicated that low FXYD2 expression was associated with poor overall survival in ccRCC patients in keeping with the previous survival analyses (Figures 4(a)–4(d), Figures S3(a)-S3(c)). Taken together, downregulated FXYD2 expression is related to poor prognosis of ccRCC, and its predictive role in prognosis can be achieved regardless of clinicopathological circumstances.

### 3.3. Relation between FXYD2 Expression and Immune Infiltration in ccRCC

**Figure 5:**
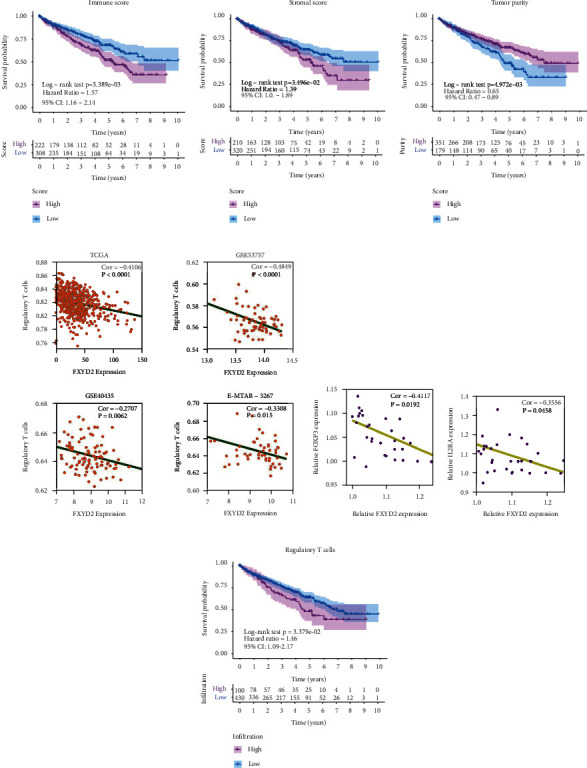
Kaplan-Meier survival analysis of ccRCC patients concerning different immune factors in the TCGA cohort. (a–c) Patients with high immune score and stromal score and low tumor purity in ccRCC had short-term overall survival. (d) FXYD2 expression negatively correlated with regulatory T cell infiltration in TCGA, E-MTAB-3267, GSE53757, and GSE40435 cohort, respectively. (e–f) Relative FXYD2 expression negatively correlated with relative Foxp3 and IL2RA expression using qPCR experiment. (g) Patients with high regulatory T cell infiltration in ccRCC had short-term overall survival.

With the assistance of the ESTIMATE algorithm, the immune and stromal scores were calculated and presented in the range of − 687.33~3339.36 and − 2115.99~5110.95, respectively, in the TCGA cohort. Derived from these two scores, the tumor purity was about 0.21 to 0.96. To clarify how these values are related to FXYD2 expression, Pearson correlation analysis revealed that immune and stromal score negatively correlated with FXYD2 expression, while tumor purity positively correlated with FXYD2 expression. Similar results were obtained after analyses in GSE40435, GSE57357, and E-MTAB-3267 cohort (Table 2). Prognosis analysis performed based on immune score, stromal score, and tumor purity indicated that patients with high immune and stromal scores and low tumor purity had worse survival (Figures 5(a)–5(c)).

The immune landscape in ccRCC was further evaluated utilizing ssGSEA in the other cohorts, which all showed negative correlations significantly between FXYD2 expression and regulatory T cell infiltration level (Figure 5(d)) except for ICGC (Figure S2(c)). This was probably related to the confounding RCC patients in the ICGC cohort mentioned above. Besides, CIBERSORT algorithm was also performed to verify the finding (Figures S4(a)-S4(e)). Foxp3 and IL2RA (CD25) are the typical markers for Treg [41, 42]. Therefore, we used our 32 clinical samples to perform qPCR experiment to validate their relation. The results revealed that the relative FXYD2 expression negatively correlated with the relative Foxp3 and IL2RA (CD25) expression (Figures 5(e)–5(f)).

FXYD2 expression had a negative correlation with checkpoint molecules, C-C chemokine receptors (CCR), and antigen-presenting cell (APC) co-inhibition (Table 2). Furthermore, Treg infiltration was classified into high and low infiltration based on optimal cut-off point from X-tile, delivering a result that patients with higher Treg infiltration would possess shorter overall survival (Figure 5(e)).

For further validating the relationship between Treg infiltration or checkpoint molecules and FXYD2 expression level in ccRCC, TIMER online database was utilized to investigate the gene markers for Treg and T cell exhaustion. About markers for Treg, CD4, IL2RA (CD25), Foxp3, CCR8, and TGF-*β* (TGFB1) inversely correlated with FXYD2 expression. As for T cell exhaustion, notable negative relationship between FXYD2 expression and CTLA-4, TIGIT, and BTLA persisted before and after tumor purity adjustment (Table 3).

### 3.4. Functional Enrichment Analysis of FXYD2 in ccRCC

**Figure 6:**
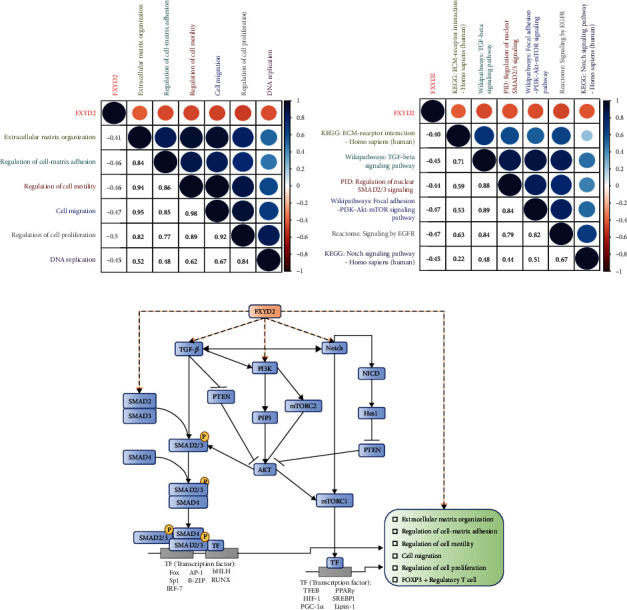
Pearson correlation analysis of FXYD2 expression and biological functions in the TCGA cohort. (a) FXYD2 expression negatively correlated with the biological functions of GO analysis in the TCGA cohort. (b) FXYD2 expression negatively correlated with the signaling pathways of KEGG analysis in the TCGA cohort. (c) Downregulated FXYD2 probably interfered with TGF-*β*-PI3K-Akt-mTOR/SMAD2/3 signaling pathway in ccRCC.

Mechanisms underlying functions of FXYD2 in ccRCC were further explored through GO and KEGG analyses. GO enrichment analysis demonstrated several biological function pathways that contribute to the possibly function of FXYD2, including extracellular matrix organization, regulation of cell-matrix adhesion, regulation of cell motility, cell migration, regulation of cell proliferation, and DNA replication (Table 4). Enriched signalling pathways of FXYD2 ECM-receptor interaction, TGF-*β* signalling, regulation of nuclear SMAD2/3 signalling, focal adhesion-PI3K-Akt-mTOR signalling, EGFR, and NOTCH signalling pathways (Table 5). Through GSVA and Pearson correlation analyses, all the biological functions and pathways inversely correlated with FXYD2 expression (Figure 6).

### 3.5. FXYD2 Protein Overexpression Suppresses Cell Proliferation, Migration, and Invasion In Vitro

**Figure 7:**
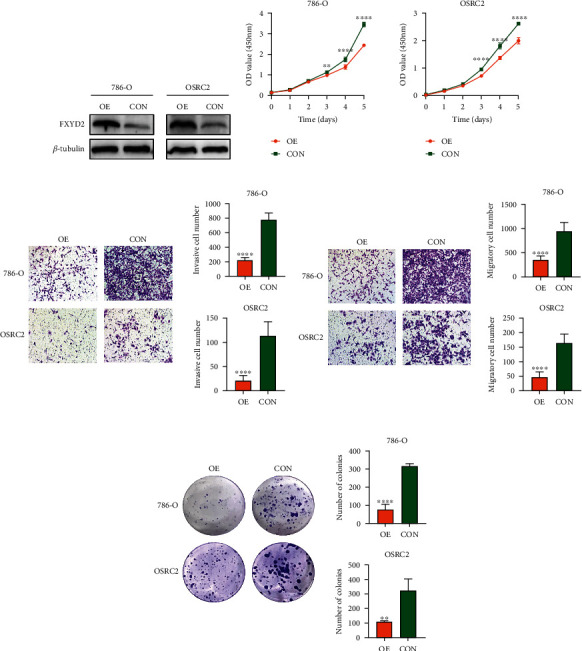
Overexpression of FXYD2 protein suppresses ccRCC cell proliferation, migration, and invasion in vitro. (a) The efficiency of FXYD2 protein overexpression was determined by WB in 786-O and OSRC2 cells. (b–e) The cell proliferation assay, migration assay, invasion assay, and colony formation assay showed that FXYD2 overexpression suppresses cell proliferation, migration, and invasion. ccRCC: clear cell renal cell carcinoma; WB: western blot; ^∗^*P* < 0.05, ^∗∗^*P* < 0.01, ^∗∗∗^*P* < 0.001, and ^∗∗∗∗^*P* < 0.0001.

Functional enrichment analysis showed that the expression of FXYD2 protein was negatively associated with regulation of cell motility, proliferation, and migration (Figure 6(a)). Therefore, we simply performed some in vitro cell experiments to validate what we found. The stably transfected 786-O and OSRC2 with FXYD2 protein overexpression were constructed, and the efficiency was determined by WB (Figure 7(a)). Then, the effect of changes in FXYD2 overexpression on the proliferation, colony formation, migration, and invasion of ccRCC cells were determined by CCK-8 assay, colony formation assay, cell migration, and invasion assays. The results showed that FXYD2 significantly inhibit cell proliferation (Figure 7(b)), colony formation (Figure 7(c)), migration (Figure 7(d)), and invasion (Figure 7(e)) in the overexpression 786-O and OSRC2 cell lines, respectively. Moreover, knockdown of FXYD2 by siRNA in above overexpression 786-O and OSRC2 cell lines showed the resecure phenomenon compared with the control groups (Figure S5).

## 4. Discussion

Approximately 14,830 people were estimated to die from renal cell carcinoma in 2020, and most were clear cell renal cell carcinoma (ccRCC) [43]. Multiple immunotherapies have been developed from nonspecific to targeted for better ccRCC management since ccRCC is immune responsive [44]. Currently, researchers spend great efforts in investigating the immune microenvironment in ccRCC not only to discover novel targets of immunotherapy but also to reduce toxicity and increase practicability [45, 46]. This study was conducted by exploring the expression and prognostic value of FXYD2 and its relationship with immune microenvironment in ccRCC to improve the practice of immunotherapy for better prognosis of ccRCC patients.

FXYD2 was identified to be significantly downregulated in ccRCC. Decreased expression of FXYD2 could independently predict worse survival and correlate with advanced tumor grade and pathologic T stage. These results indicated that FXYD2 may act as a suppressor tumor gene in ccRCC. FXYD family contains seven single-span transmembrane proteins modulating the properties of Na^+^/K^+^-ATPase [47]. Genes encoding these proteins were shown to participate in carcinogenesis and were able to act as prognostic predictors of tumors [48–50]. As a member of this family, FXYD2 takes part in dealing with cancer. Study demonstrated FXYD2 was a therapeutic target in ovarian clear cell carcinoma [20]. It also involved in the metastasis of osteosarcoma [51]. Meanwhile, Na^+^/K^+^-ATPase, the major functional target of FXYD2, was discovered to be a critical transducer and integrator of signal involved in carcinogenesis and was investigated as a therapeutic target [52]. Additionally, a pan-cancer analysis using TIMER2.0 online tool showed that FXYD2 mRNA expression was also downregulated in bladder cancer, breast cancer, kidney renal papillary cell carcinoma, liver cancer, lung squamous cell carcinoma, prostate cancer, and thyroid cancer, while it was upregulated in cholangiocarcinoma (*P* < 0.05, Figure S1(h)). However, how FXYD2 works as a prognostic predictor in ccRCC requires further investigation.

With the help of the ESTIMATE algorithm, FXYD2 was revealed to conversely correlate with immune and stromal scores and positively related to tumor purity which was determined by the percentage of infiltrating immune or stromal cells. High immune scores and stromal scores and low tumor purity were connected to short-term overall survival of ccRCC patients. These results indicated that the function of FXYD2 may correlate with increased nontumor components in ccRCC. Among the cancer mass, immune cells are elucidated to be the major component [53, 54]. Different tumor-infiltrating immune cells have different effects on tumor cells, such as Treg potentiate tumor growth, while CD8^+^ T cells improve tumor prognosis [29]. In the kidney, FXYD2 functions to control the activity of Na^+^/K^+^-ATPase, which appears to have properties of regulating the activation of T cell proliferation [55]. Thus, the prognostic effect of FXYD2 may correlate with the increased immune component in ccRCC.

To further investigate the FXYD2-related immune involvement in ccRCC, ssGSEA was conducted to demonstrate that decreased FXYD2 expression significantly increased enrichment of Treg, checkpoint, CCR, and APC co-inhibition. Treg are a subtype of T lymphocytes which are discovered to be a critical participant in immune tolerance and able to suppress antitumor immunity in variable cancers with worse prognosis [56–58]. As indicated by the TIMER database, this type of cell is CD4^+^CD25^+^FOXP3^+^ in which FOXP3 (Forkhead box P3) is the major regulatory transcription factor modulating the suppressive effects of Treg [59, 60]. According to the in-depth research, Treg can inhibit CD8^+^ effector T cells, which are the major defender to tumor, with the help of multiple functional surface molecules and inhibitory cytokines like TGF-*β* (transforming growth factor-*β*) [61, 62]. A considerable portion of the suppressive function of Treg is mediated through TGF-*β* which is also critical for Treg induction through modulating FOXP3 expression [63, 64]. Immune checkpoints are known as the manager of lymphocytes activation. Increased immune checkpoint molecules were identified in tumors, leading to a decreased function of T cells and thus the antitumor immunity [65]. CTLA-4 (cytotoxic T lymphocyte antigen-4), TIGIT (T cell immunoreceptor with immunoglobulin and immunoreceptor tyrosine-based inhibitory motif domain), and BTLA (B and T lymphocyte attenuator) are inhibitory receptors acting as immune checkpoints in T lymphocytes. All of them are not only responsible for T cells inactivation and exhaustion in the tumor microenvironment but also related to Treg function [66–68]. In recent years, immune checkpoint inhibitors targeting Treg attracted great interest in the field of ccRCC treatment, and some of them had been proven to be promising cancer therapeutic targets [69]. C-C chemokine receptors (CCR) and G protein-coupled receptors locate in the cell membrane of lymphocytes, and they combine with chemokines to regulate cell proliferation, activation, differentiation, extracellular matrix remodeling, angiogenesis, and tumor metastasis [70, 71]. Among this family, CCR8 was discovered to be increased in Treg. Interacting with its cognate ligand CCL1, CCR8 induced Treg migration and retention in the tumor microenvironment [72, 73]. APCs, as the name indicated, play a role in presenting antigens to immune cells for subsequent reactions. In the tumor microenvironment, to some extent, APCs were found to have suppressed function and possess potential effects in inhibiting antitumor T cells [74]. Taken together, downregulated FXYD2 could probably correlate with increased activation and migration of Treg which suppress effector T cells and thus hamper antitumor immunity. The list of molecules involved in this process is worth further exploration with the concern of providing novel biomarkers and enhancing existing immunotherapies.

GO analysis demonstrated that FXYD2 expression negatively correlates with extracellular matrix organization, regulation of cell-matrix adhesion, regulation of cell motility, cell migration, and regulation of cell proliferation. Our laboratory experiments validated the findings above. These biological functions meet with the immune involvement of FXYD2 in ccRCC, which suggests the role of FXYD2 in the recruitment and activation of Treg. Meanwhile, KEGG analysis provided several potential signaling pathways like ECM-receptor interaction, TGF-*β* signaling pathway, regulation of nuclear SMAD2/3 signaling, focal adhesion-PI3K-Akt-mTOR-signalling pathway, signaling by EGFR, and NOTCH signaling pathway. SMAD2 and SMAD3 belong to the important SMAD family proteins, which are downstream proteins in TGF-*β* signaling pathway, inducing and maintaining FOXP3 expression. TGF-*β* signaling pathway modulates the differentiation and function of Treg through SMAD2/3 signaling intracellularly [75]. Drugs targeting TGF-*β*-SMAD2/3 pathway were proven to have suppressive effect on the growth and invasion of cancer cells [76]. The PI3K (phosphatidylinositol 3-kinase)-Akt (protein kinase B) and mTOR (mammalian target of rapamycin) pathways appear as two vital intracellular signaling pathways with multiple physiological and pathological functions. However, in some circumstances, they cooperate with each other resembling one signaling pathway in the cell cycle regulation [77]. PI3K-Akt-mTOR signaling pathway has been found to be activated in various tumors, and it attracts great attention for immunotherapeutic research as some inhibitors for it has been approved to be safe and effective in the clinic [78]. Similar to SMAD2/3 signaling pathway, PI3K-Akt-mTOR signaling pathway was revealed to regulate the expression of FOXP3 in Treg [79]. Meanwhile, PI3K-Akt can cross talk with TGF-*β* signaling pathway in a SMAD-dependent manner at late stage of tumorigenesis, while some researchers demonstrated the positive function of TGF-*β*-PI3K-AKT-mTOR signaling pathway in tumor progression [80–82]. NOTCH signaling pathway regulates a wide range of cell fate throughout development and homeostasis, and its functions vary in different tissue [83]. Activation of NOTCH pathway was identified in ccRCC, and inhibitors targeting this pathway led to suppression of the cancer cells [84]. The oncogenic role of NOTCH pathway was depicted that not only it was mediated by PI3K-Akt signaling pathway but also it could be achieved through mTOR directly [85, 86]. Additionally, NOTCH pathway was revealed to play a positive role in regulating antitumor cytotoxic T cells activation and was related to Treg [87]. Moreover, NOTCH signaling pathway could interact with TGF-*β* signaling through downstream protein-protein interaction [88]. Therefore, taken our results and previous findings together, the function of FXYD2 expression in the prognosis and immune cell infiltration in ccRCC may correlate with TGF-*β*-SMAD2/3, Notch, and PI3K-Akt-mTOR signaling pathways (Figure 6).

In the current study, FXYD2 was identified as a novel prognostic biomarker for ccRCC, shedding light on the immune microenvironment in ccRCC. The mechanism underlying Treg infiltration in ccRCC and the potential signaling pathways provide insights for further investigation of immunotherapies. Nonetheless, there are still some limitations in this study. Most of the data utilized in this research were retrieved from public datasets with a potential defect of data affecting the accuracy of results although there were a few clinical tissue samples and in vitro functional experiment used for validating the expression level and biological behavior influenced by FXYD2 protein. Meanwhile, the in vivo biological behavior and mechanisms proposed in this study require further experiments for validation and investigation.

## 5. Conclusion

In summary, the present study comprehensively analyzed FXYD2 expression in ccRCC utilizing several public datasets and our clinical tissue samples and demonstrated that downregulated FXYD2 expression correlated with carcinogenesis, poor prognosis, and increased Treg infiltration in ccRCC, which may function by participating in TGF-*β*-SMAD2/3, Notch, and PI3K-Akt-mTOR signaling pathways. These findings probably provide a novel prognostic marker and potential therapeutic targets for further investigation of ccRCC.

## Figures and Tables

**Table 1:** Chi-square test of FXYD2 expression and clinicopathological factors in the TCGA cohort.

Parameters	FXYD2		*P* value
	Low (*n* = 82)	High (*n* = 448)	
Age			0.155
< 60	32	213	
≥ 60	50	235	
Sex			0.485
Female	26	160	
Male	56	288	
Race			0.744
Asian	2	6	
Black	9	47	
White	71	395	
Smoking			0.847
No	45	251	
Yes	37	197	
Radiation			0.935
No	81	1	
Yes	443	5	
Pharmaceutical			0.891
No	70	385	
Yes	12	63	
Grade			**0.016**
1	0	14	
2	31	196	
3	29	179	
4	20	55	
x	2	4	
Stage			0.151
1	32	235	
2	10	47	
3	23	101	
4	17	65	
T			**0.001**
1	33	238	
2	10	59	
3	33	146	
4	6	5	
M			0.073
M0	65	355	
M1	16	62	
Mx	1	31	
N			0.208
N0	36	203	
N1	5	11	
Nx	41	234	

**Table 2:** Relation between FXYD2 expression and immune landscape in ccRCC based on ssGSEA and ESTIMATE in TCGA, GSE40435, GSE57357, and E-MTAB-3267 cohort.

Algorithm	Terms	TCGA		GSE40435		GSE57357		E-MTAB-3267	
		Cor	*P* value	Cor	*P* value	Cor	*P* value	Cor	*P* value
ssGSEA	Checkpoint	− 0.344	<0.0001	− 0.47	<0.0001	− 0.537	<0.0001	− 0.303	0.0197
	CCR	− 0.407	<0.0001	− 0.442	<0.0001	− 0.558	<0.0001	− 0.329	0.011
	APC co-inhibition	− 0.219	<0.0001	− 0.464	<0.0001	− 0.299	0.011	− 0.439	0.0005
ESTIMATE	Tumor purity	0.392	<0.0001	0.447	<0.0001	0.292	0.0129	0.423	0.0008
	Immune score	− 0.295	<0.0001	− 0.411	<0.0001	− 0.231	0.0512	− 0.342	0.008
	Stromal score	− 0.489	<0.0001	− 0.393	<0.0001	− 0.28	0.0173	− 0.477	0.0001

**Table 3:** Correlation analysis between FXYD2 and gene markers of regulatory T cells and T cell exhaustion in the TIMER database.

Description	Gene markers	KIRC			
		None		Purity	
		Cor	*P* value	Cor	*P* value
Regulatory T cells	CD4	− 0.288003913	1.22E-11	− 0.239189152	2.02E-07
	IL2RA (CD25)	− 0.526918232	2.06E-39	− 0.51734476	6.43E-33
	Foxp3	− 0.300129041	1.48E-12	− 0.260752041	1.33E-08
	CCR8	− 0.30884178	3.04E-13	− 0.295972971	8.97E-11
	TGF-*β* (TGFB1)	− 0.440139103	1.16E-26	− 0.391507329	2.45E-18
	STAT5B	− 0.074059486	0.087612567	− 0.069482957	0.136326049

T cell exhaustion	CTLA-4	− 0.16977005	8.19E-05	− 0.125303249	0.007066482
	PD-1 (PDCD1)	− 0.03253211	0.453557607	0.005050198	0.913885703
	TIM-3 (HACVR2)	0.071778106	0.097849172	0.106003996	0.022831654
	LAG3	− 0.107790852	0.012775711	− 0.061425448	0.187998507
	TIGIT	− 0.212794306	7.12E-07	− 0.18434193	6.85E-05
	BTLA	− 0.264015494	5.97E-10	− 0.235523261	3.13E-07

**Table 4:** GO analysis of FXYD2 in the TCGA cohort.

GO terms	*P* value	Related genes
Extracellular matrix organization	2.21E-12	COLGALT1; COL11A1; ITGA5; ADAM12; ADAM19; COL3A1; SERPINE1; LRP1
		LOXL2; PLOD2; FAP; PTX3; CD44; FBN1; COL5A2; POSTN; COL5A1;
		COL6A3; FN1; COL1A2; COL12A1; COL1A1; TGFBI; SERPINH1; PXDN
Regulation of cell-matrix adhesion	0.014361	POSTN; LRP1; SERPINE1; PLAU; MAP4K4
Regulation of cell motility	0.001664	MAP4K4; ITGA5; MSN; FBXO5; ADAM17; COL3A1; ABL2; SERPINE1; IL1R1; CCSAP; SRPX2; DPYSL3; PLAU; POSTN; SNAI2; LRP1; FN1; GTSE1; F2R; RND3; GNA12; COL1A1
Cell migration	1.36E-05	MAP4K4; ITGA5; MSN; ADAMTS12; FBXO5; ADAM17; COL3A1; SERPINE1; IL1R1; SHC1; LOXL2; CDK1; CCDC88A; FAP; NAV1; GLI3; AXL; SRPX2; HRH1; GNA12; FAM83D; FBXO45; DPYSL3; CD44; ANLN; PLAU; POSTN; COL5A1; SNAI2; ASPM; LRP1; FN1; GTSE1; F2R; RND3; COL1A2; COL1A1; CTHRC1
Regulation of cell proliferation	0.001497	CCNB2; CTC1; FOXM1; RAPGEF1; ADAM17; PRC1; ABL2; GNAI3; CDC7; CCNA2; CDK1; CDKN3; CCDC88A; FAP; CCNF; GLI3; OSMR; CDCA7; ASPM; SHC1; KMT2D; PLAU; SNAI2; CHST11; IL2RA; SHCBP1; FN1; RUNX2; F2R; CDK2; NAP1L1; PRRX1; CTHRC1; FBXO5
DNA replication	3.19E-07	FBXO5; GINS1; CCNA2; CDK1; CCDC88A; EXO1; DSCC1; MSH6; MCM6; CDK2; RBMS1; RRM2; NAP1L1; CTC1; WDHD1; CDC7

**Table 5:** KEGG analysis of FXYD2 in the TCGA cohort.

Pathway terms	P-value	Related genes
KEGG: ECM-receptor interaction-Homo sapiens (human)	2.96E-05	HMMR; FN1; CD44; THBS2; ITGA5; COL1A2; COL1A1; COL6A3
WikiPathways: TGF-*β* signaling pathway	0.003643	CCNB2; SHC1; FN1; RUNX2; CDK1; COL1A2; SKIL
RID: Regulation of nuclear SMAD2/3 signaling	0.001009	RUNX2; CDK2; RUNX1; COL1A2; SKI; SERPINE1
WikiPathways: Focal adhesion-PI3K-Akt-mTOR-signalling pathway	0.000177	IL2RA; COL11A1; FN1; SLC2A3; THBS2; ITGA5; F2R; GNB4; COL5A2; OSMR; COL1A2; COL1A1; COL3A1; COL5A1
Reactome: Signaling by EGFR	0.003773	PTPN12; ADAM12; SHC1; ADAM17
KEGG: NOTCH signaling pathway-Homo sapiens (human)	0.034696	MAML1; NOTCH2; ADAM17

## Data Availability

The data and information demonstrated and analyzed throughout the present study were obtained from The Cancer Genome Atlas (TCGA, http://cancergenome.nih.gov), ArrayExpress (https://www.ebi.ac.uk/arrayexpress), Genome Expression Omnibus (GEO, https://www.ncbi.nlm.nih.gov/geo), ICGC (https://dcc.icgc.org/), The Human Protein Atlas Project (https://www.proteinatlas.org), TIMER (Tumor IMmune Estimation Resource, https://cistrome.shinyapps.io/timer), and ConsensusPathDB (http://cpdb.molgen.mpg.de/).

## Abbreviation

ccRCC: Clear cell renal cell carcinoma
Treg: Regulatory T cells
TCGA: The Cancer Genome Atlas
GEO: Genome Expression Omnibus
RT-PCR: Quantitative real-time polymerase chain reaction
WB: Western blot
IHC: Immunohistochemistry
OS: Overall survival
PFS: Progression-free survival
DSS: Disease-specific survival
DFS: Disease-free survival
ESTIMATE: Estimation of Stromal and Immune cells in Malignant Tumor tissue using Expression data
ssGSEA: Single-sample Gene Set Enrichment Analysis
TIMER: Tumor IMmune Estimation Resource
GO: Gene ontology
KEGG: Kyoto Encyclopedia of Genes and Genomes
tROC: Time-dependent receiver operating characteristic curve.

## Supplementary Materials

Supplementary 1 Appendix 1: Figure S1. Expression patterns of FXYD2. (a–e) The expression level of FXYD2 in different tumor grade, cancer stages, nodal metastasis status, patient's race, and patient's gender, respectively. (f–g) IHC images from the Human Protein Atlas. (h) The pan-cancer analysis for the expression level of FXYD2.

Supplementary 2 Appendix 2: Figure S2. Prognostic values of FXYD2. (a) Time-dependent ROC analysis for the FXYD2 expression in TCGA cohort. (b) The survival analysis of RCC patients based on FXYD2 expression in ICGC cohort. (c) The correlation analysis between FXYD2 expression and Treg infiltration in RCC samples from ICGC cohort. ROC: receiver operating characteristic curve; Treg: regulatory T cells.

Supplementary 3 Appendix 3: Figure S3. Kaplan-Meier survival analysis of FXYD2 concerning different clinicopathological factors. (a) Either patient at stages 1-2 or stages 3-4 had short-term overall survival when their FXYD2 expression was low. (b) Either patient at grades 1-2 or grades 3-4 had short-term overall survival when their FXYD2 expression was low. (c) Either patient with or without metastasis had short-term overall survival when their FXYD2 expression was low.

Supplementary 4 Appendix 4: Figure S4. Relation between FXYD2 expression and Treg infiltration using CIBERSORT algorithm in TCGA (a), GSE40435 (b), GSE53757 (c), E-MATB-3267 (d), and ICGC (e).

Supplementary 5 Appendix 5: Figure S5. FXYD2 knockdown in two overexpression cell lines suppresses cell proliferation, migration and invasion in vitro. (a) The efficiency of FXYD2 expression knockdown was determined by qPCR in 786-O and OSRC2 cell lines. (b–e) The cell proliferation, migration, invasion and colony formation were inhibited in FXYD2 knockdown cell lines. ^∗^*P* < 0.05, ^∗∗^*P* < 0.01, ^∗∗∗^*P* < 0.001, and ^∗∗∗∗^*P* < 0.0001.
