# Supplementary figures and images for: Downregulation of FXYD2 Is Associated with Poor Prognosis and Increased Regulatory T Cell Infiltration in Clear Cell Renal Cell Carcinoma
Source: J Immunol Res. 2022 Oct 19;2022:4946197. doi: 10.1155/2022/4946197 (PMC9606837; doi:10.1155/2022/4946197)

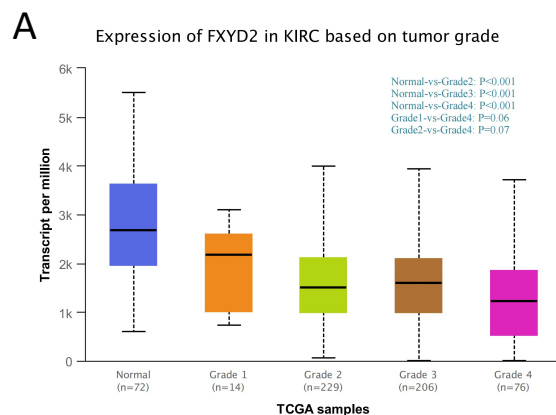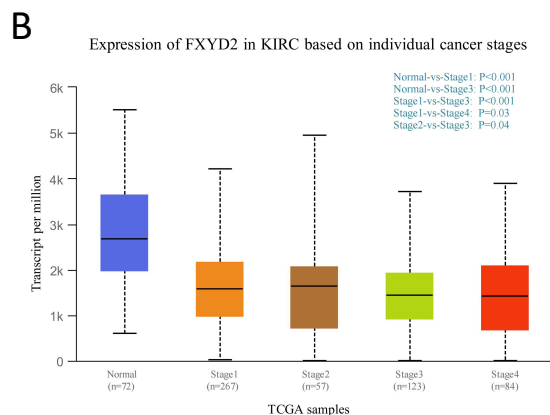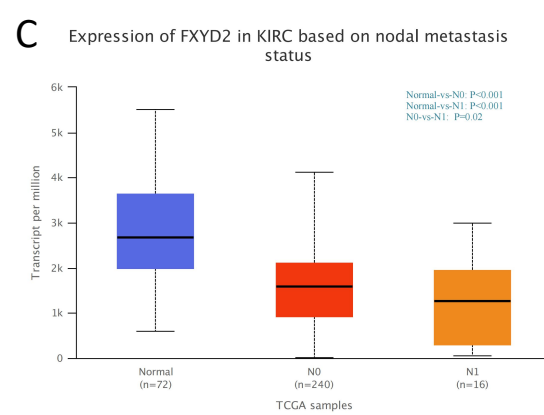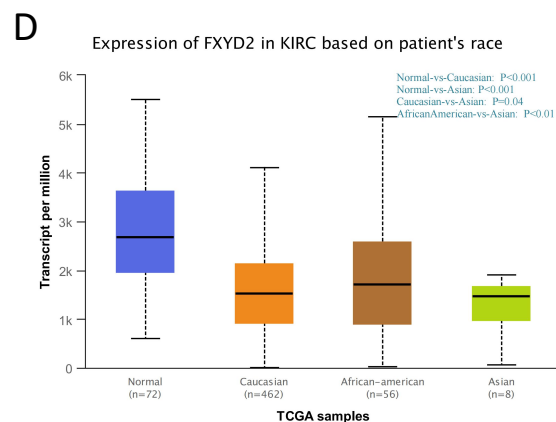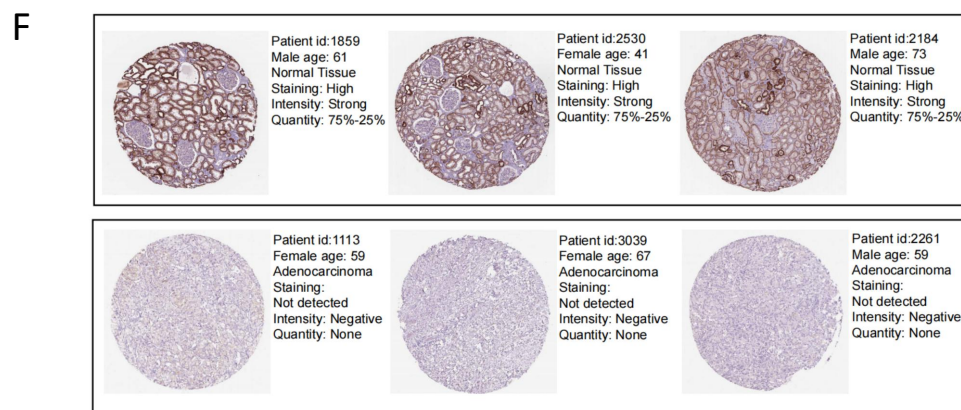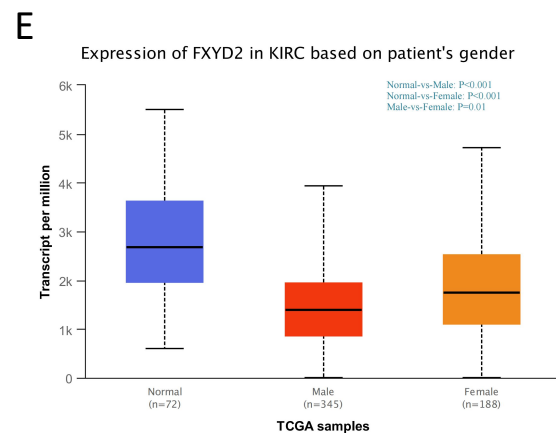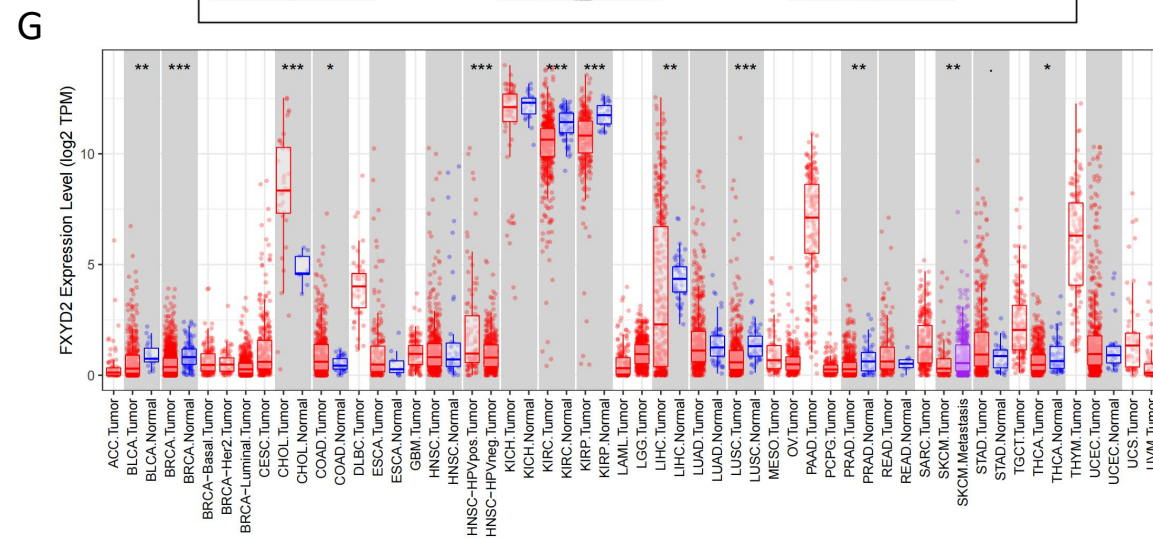

Supplement: Supplementary 1 — Appendix 1: Figure S1. Expression patterns of FXYD2. (a–e) The expression level of FXYD2 in different tumor grade, cancer stages, nodal metastasis status, patient's race, and patient's gender, respectively. (f–g) IHC images from the Human Protein Atlas. (h) The pan-cancer analysis for the expression level of FXYD2. [file 4946197.f1.pdf]

A

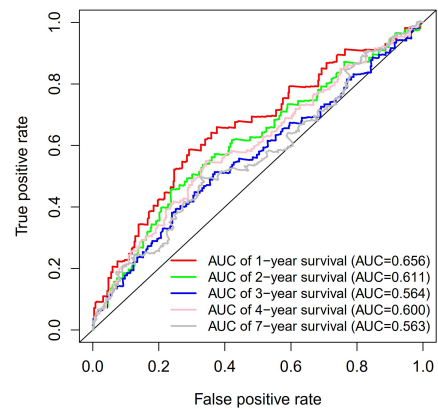

B

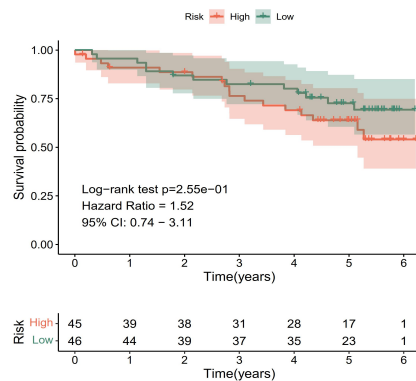

C

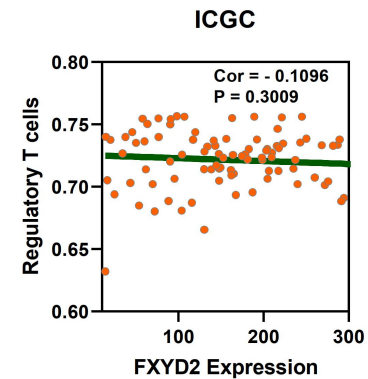

Supplement: Supplementary 2 — Appendix 2: Figure S2. Prognostic values of FXYD2. (a) Time-dependent ROC analysis for the FXYD2 expression in TCGA cohort. (b) The survival analysis of RCC patients based on FXYD2 expression in ICGC cohort. (c) The correlation analysis between FXYD2 expression and Treg infiltration in RCC samples from ICGC cohort. ROC: receiver operating characteristic curve; Treg: regulatory T cells. [file 4946197.f2.pdf]

A

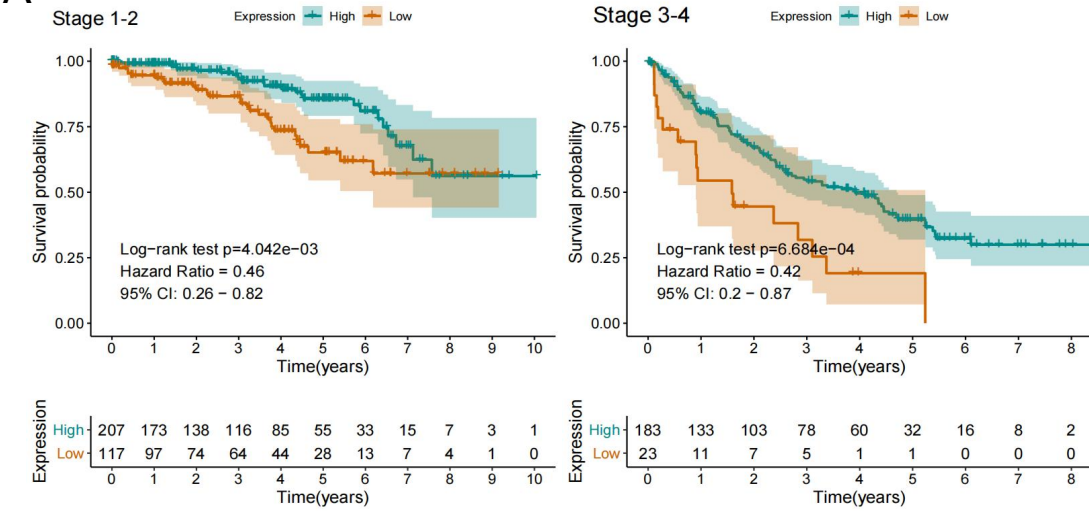

B

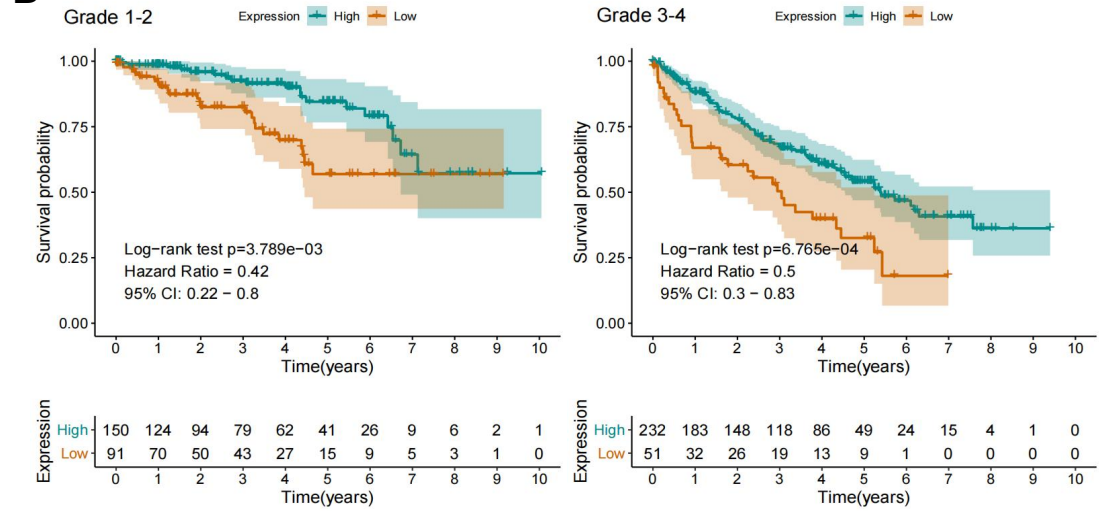

C

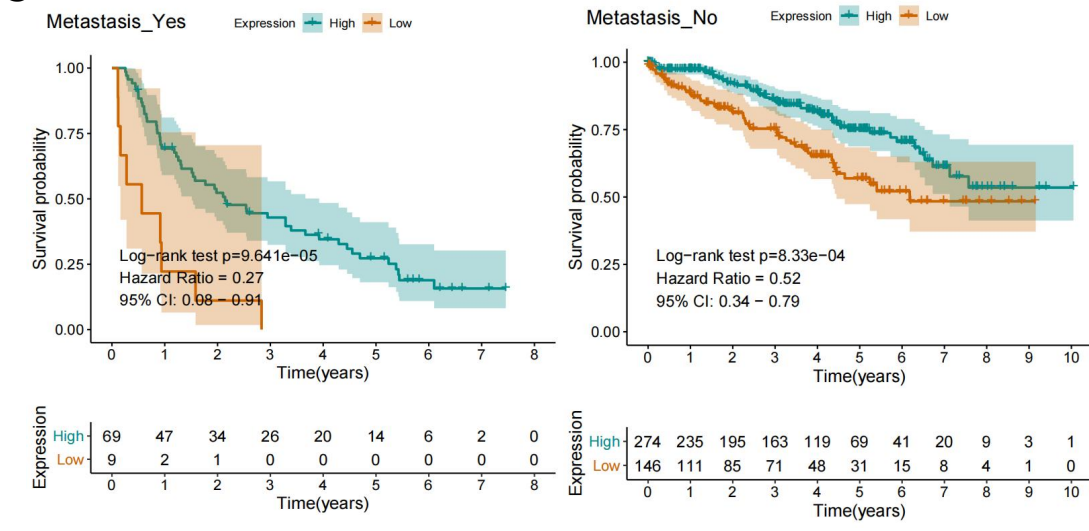

Supplement: Supplementary 3 — Appendix 3: Figure S3. Kaplan-Meier survival analysis of FXYD2 concerning different clinicopathological factors. (a) Either patient at stages 1-2 or stages 3-4 had short-term overall survival when their FXYD2 expression was low. (b) Either patient at grades 1-2 or grades 3-4 had short-term overall survival when their FXYD2 expression was low. (c) Either patient with or without metastasis had short-term overall survival when their FXYD2 expression was low. [file 4946197.f3.pdf]

A

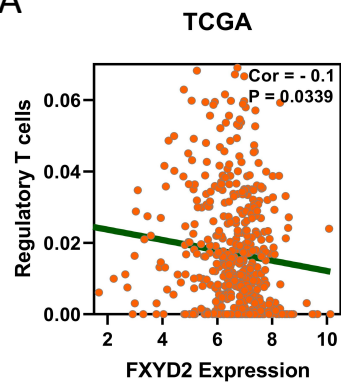

B

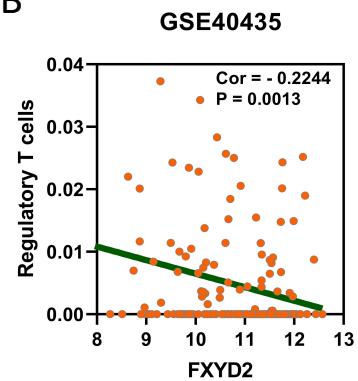

C

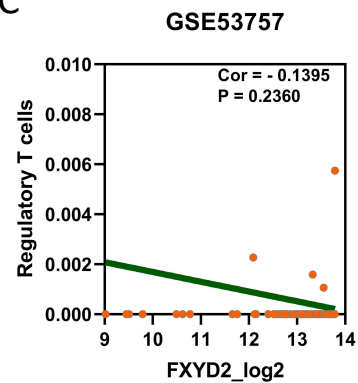

D

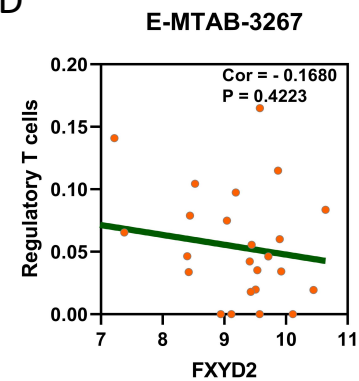

E

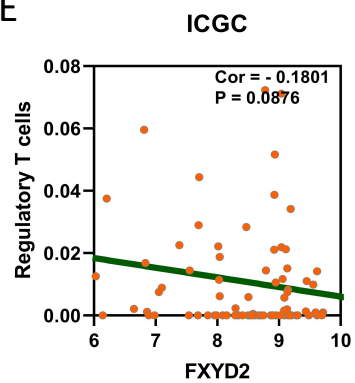

Supplement: Supplementary 4 — Appendix 4: Figure S4. Relation between FXYD2 expression and Treg infiltration using CIBERSORT algorithm in TCGA (a), GSE40435 (b), GSE53757 (c), E-MATB-3267 (d), and ICGC (e). [file 4946197.f4.pdf]

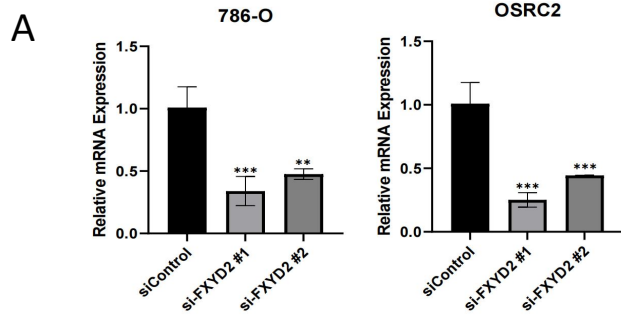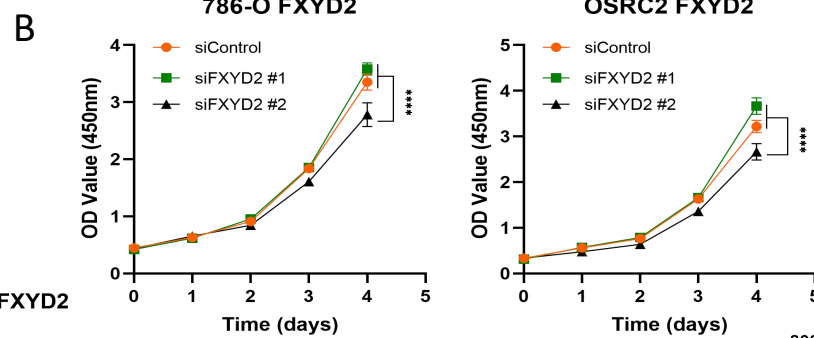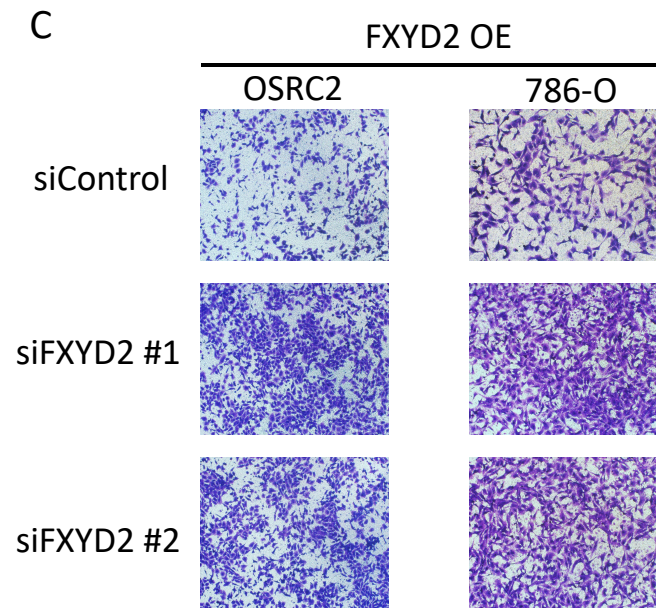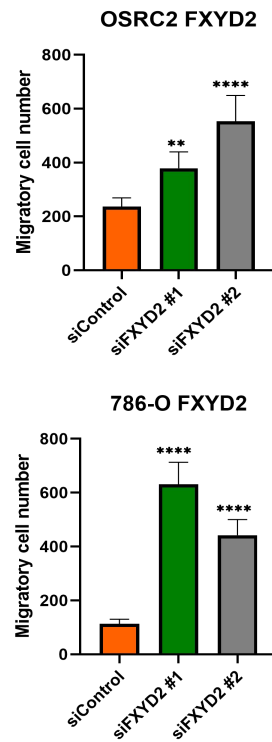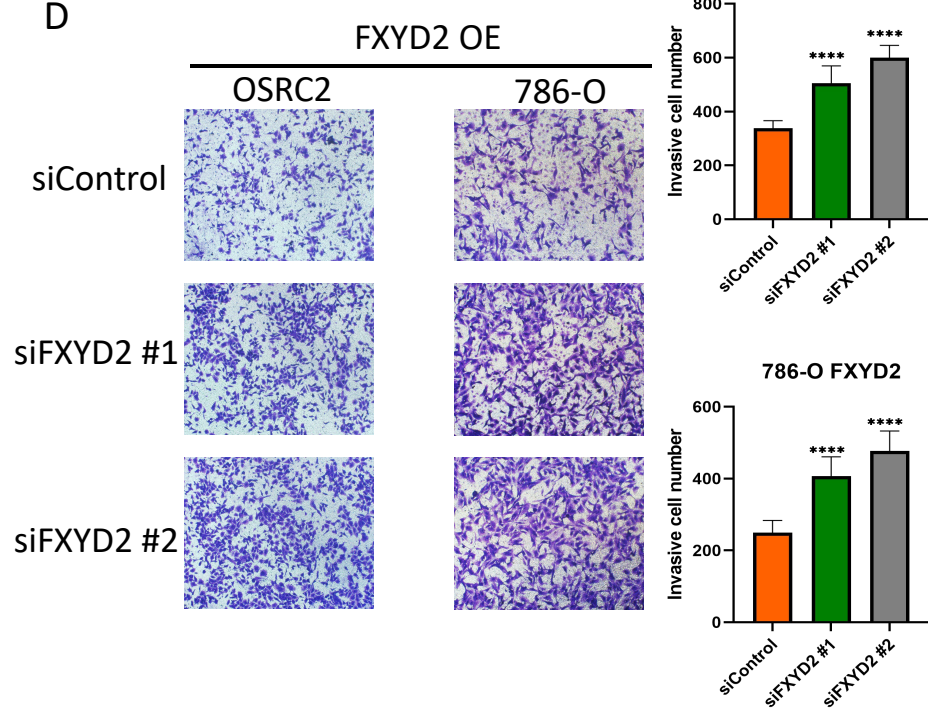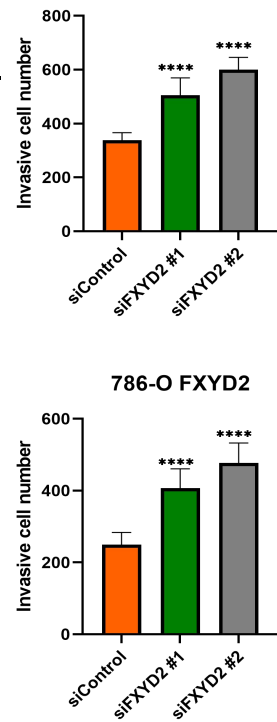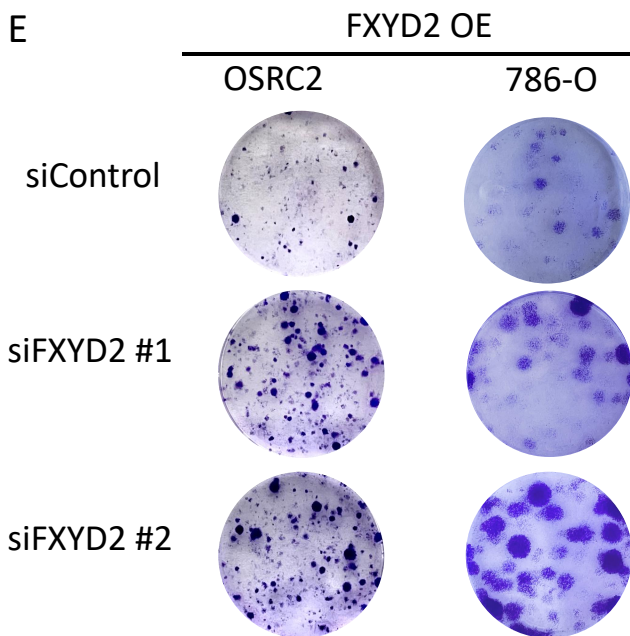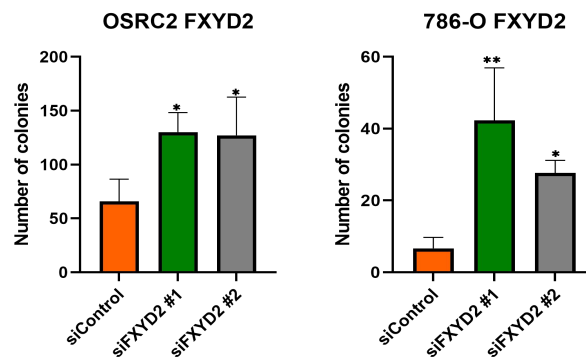

Supplement: Supplementary 5 — Appendix 5: Figure S5. FXYD2 knockdown in two overexpression cell lines suppresses cell proliferation, migration and invasion in vitro. (a) The efficiency of FXYD2 expression knockdown was determined by qPCR in 786-O and OSRC2 cell lines. (b–e) The cell proliferation, migration, invasion and colony formation were inhibited in FXYD2 knockdown cell lines. ∗P < 0.05, ∗∗P < 0.01, ∗∗∗P < 0.001, and ∗∗∗∗P < 0.0001. [file 4946197.f5.pdf]
